# Supplementary material for: Learning curve and surgical outcome of robotic assisted colorectal surgery with ERAS program
Source: Sci Rep. 2022 Nov 29;12:20566. doi: 10.1038/s41598-022-24665-w (PMC9709162; doi:10.1038/s41598-022-24665-w)
Supplement: Supplementary file 1 — Supplementary Information 1. [file 41598_2022_24665_MOESM1_ESM.zip › LSC Raw data-Ñ[▒K/128/N_ñw╝╨░O▒Kñσ.pdf]

W52 / W52

臺中榮民總醫院
手術室護理記錄

[redacted]

基本資料

- 室別： 開刀房-06室，手術科別： CRS，手術類別： 常規，日期： 2021/06/07
- 進入時間： 1120 ； 麻醉開始時間： 1120 ； Time Out時間： 1245 ； 劃刀時間： 1246 ； 手術結束時間： 1715 送出時間： 1740 ；
- 麻醉方式： GE
  診斷：Adenomatous polyposis syndrome
  手術名稱：1.Laparoscopic right colectomy and anastomosis2.Low anterior resection +/- pelvic LN dissection3.4.
  手術局部用藥：NIL
  術前預防性抗生素：
  - 抗生素名稱一： Veterin 2GM IVD/由麻醉護理人員給予
  - 時間： 1230
  - 上次簽章: 陳昱穎

  ，追加預防性抗生素：
- 手術醫師： 蔣鋒帆，林陳彥，古朋育，洪浚嚴
- 麻醉醫師： VS 李淑娟
-
  - 刷手護士A ： 許雅芳，時間： 1120，接替刷手一： 陳昱穎，時間： 1200，接替刷手二： 許雅芳，時間： 1230
  - 流動護士A ： 羅媗鈴，時間： 1120，接替流動一： 陳昱穎，時間： 1230，接替流動二： 羅媗鈴，時間： 1300
- 更多接替及流動護士： 接替刷手三： 陳昱穎，時間： 1500，接替流動三： 彭芳渝，時間： 1500，接替流動四： 張?文，時間： 1650，接替流動五： 彭芳渝，時間： 1725

皮膚狀況

A-V Shunt： 無
術前： 完整
記錄者： 上次簽章: 陳昱穎
術後： 完整
記錄者： 彭芳渝

手術準備

手術臥式 單一手術臥式

-
  - 手術臥式： 截石臥式
  - 使用附件： 溫毯 38 ℃，腳架
  - 皮膚準備： 10%Aq Betadine，Hibiscrub，2% Chlorhexidine
  - 電燒： 使用電燒: 電極片位置： 其它： 迴路電極墊，記錄者： 上次簽章: 陳昱穎

止血帶T： 無

植入物

- 植入物名稱/號碼/數量/部位：Ethicon Endo GIA 60-4.1mmx3釘/Rectum
  60-3.5mmx1釘/迴腸

，記錄者： 上次簽章: 彭芳渝

標本送檢

- 細菌培養 無
- 細胞學 無
- 冰凍切片 無
- 病理標本
  組織名稱一：colon+rectum
- 截肢 無

標本交付 標本： 1 袋，標本： 0 盒，標本： 0 瓶，細胞學： 0 管，Culture Tube： 0 管，被交付醫師： R 古朋

育，

- 記錄者： 上次簽章: 彭芳渝
- 時間： 1640

傷口術後敷料

傷口敷料：

- 請於圖上標示部位：

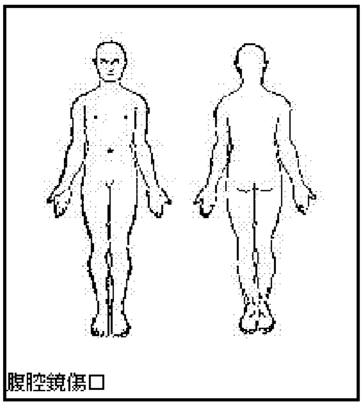

- 傷口敷料： menpore覆蓋

導管

導管	天數	導管資訊	插入日期	插入時間	更換日期	拔除日期	拔除時間	拔除原因	預計拔除/更換	預計拔除/更換日期
A-line (1)	1	號碼:20 號; 部位:左手;	2021/06/07	1230						
Penrose Drain(1)	1	號碼:12 mm.; 部位:骨盆腔;	2021/06/07	1625						
Foley(1)	1	號碼:16 Fr.; 部位:膀胱;	2021/06/07	1145						
Sump NG Tube(1)	1	號碼:16 Fr.; 部位:胃; 註記:fix 65 cm;	2021/06/07	1225						

用物計數

- 手術中出血量： minimal c.c.
- 手術中尿量： 500 c.c.
-
    - 其它： 無

用物計數： 多切口同時縫合

- 1.器械： 正確，2.縫針： 正確，3.刀片： 正確，4.紗布： 正確，5.腹部墊： 未使用，6.紗球： 未使用，7.PEANUTS ： 未使用，8.紗條： 未使用，9.絲帶： 未使用，10.針頭： 未使用，11.Vessel loop： 未使用，12.Boots： 未使用，13.Jelco： 未使用，14.棉條： 未使用，
    - 第一次：
        - 刷手護士 ： 陳昱穎
        - 流動護士： 上次簽章: 彭芳渝
        - 時間： 1630

    ,
    - 第二次：
        - 刷手護士 ： 陳昱穎
        - 流動護士： 上次簽章: 彭芳渝
        - 時間： 1640

    ,
    - 第三次：
        - 刷手護士 ： 陳昱穎
        - 流動護士： 上次簽章: 彭芳渝
        - 時間： 1645

備註

備註： olympus腹腔鏡 NO.5

- 記錄者： 上次簽章: 陳昱穎
- 時間： 1258

填寫護理人員 彭芳渝

正式摘要

W52 / W52

doctorRank:B
